# Lipoprotein Particle Profiles Associated with Telomere Length and Telomerase Complex Components

**DOI:** 10.3390/nu15112624

**Published:** 2023-06-03

**Authors:** Nil Novau-Ferré, Melina Rojas, Laia Gutierrez-Tordera, Pierre Arcelin, Jaume Folch, Christopher Papandreou, Mònica Bulló

**Affiliations:** 1 Nutrition and Metabolic Health Research Group, Department of Biochemistry and Biotechnology, Rovira i Virgili University (URV), 43201 Reus, Spain; nil.novau@urv.cat (N.N.-F.); melinaisabella.rojas@urv.cat (M.R.); laia.gutierrez@iispv.cat (L.G.-T.); jaume.folch@urv.cat (J.F.); 2 Institute of Health Pere Virgili (IISPV), 43204 Reus, Spain; pierrealphonse.arcelin@salutsantjoan.cat; 3 Center of Environmental, Food and Toxicological Technology(TecnATox), Rovira i Virgili University, 43201 Reus, Spain; 4 Atención Básica de Salud (ABS) Reus V. Centro de Atención Primaria Marià Fortuny, SAGESSA, 43204 Reus, Spain; 5 Biomedical Research Networking Centre in Neurodegenerative Diseases (CIBERNED), Carlos III Health Institute, 28031 Madrid, Spain; 6 Department of Nutrition and Dietetics Sciences, School of Health Sciences, Hellenic Mediterranean University (HMU), 72300 Siteia, Greece; 7 CIBER Physiology of Obesity and Nutrition (CIBEROBN), Carlos III Health Institute, 28029 Madrid, Spain

**Keywords:** lipoprotein subclasses, telomere length, telomerase complex components, *TERT*, *WRAP53*

## Abstract

Telomere length (TL) is a well-known marker of age-related diseases. Oxidative stress and inflammation increase the rate of telomere shortening, triggering cellular senescence. Although lipoproteins could have anti-inflammatory and proinflammatory functional properties, the relationship between lipoprotein particles with TL and telomerase activity-related genes has not been investigated much. In this study, we assessed the associations of lipoprotein subfractions with telomere length, *TERT*, and *WRAP53* expression in a total of 54 pre-diabetic subjects from the EPIRDEM study. We regressed TL, *TERT*, and *WRAP53* on 12 lipoprotein subclasses, employing a Gaussian linear regression method with Lasso penalty to determine a lipoprotein profile associated with telomere-related parameters. The covariates included age, sex, body mass index (BMI), dyslipidemia, statin consumption, and physical activity leisure time. We identified a lipoprotein profile composed of four lipoprotein subfractions associated with TL (Pearson r = 0.347, *p*-value = 0.010), two lipoprotein subfractions associated with *TERT* expression (Pearson r = 0.316, *p*-value = 0.020), and five lipoprotein subfractions associated with *WRAP53* expression (Pearson r = 0.379, *p*-value =0.005). After adjusting for known confounding factors, most lipoprotein profiles maintained the association with TL, *TERT*, and *WRAP53*. Overall, medium and small-sized HDL particles were associated with shorter telomeres and lower expression of *TERT* and *WRAP53*. Large HDL particles were associated with longer telomere and lower expression of *WRAP53*, but not with *TERT*. Our results suggest that the lipoprotein profiles are associated with telomere length, *TERT*, and *WRAP53* expression and should be considered when assessing the risk of chronic diseases.

## 1. Introduction

Aging is associated with a progressive decline in several physiological processes, leading to an increased risk for several non-communicable diseases including cardiometabolic diseases and cancer. Telomere length is nowadays considered one of the best biomarkers of aging, and its shortening is a well-known marker in age-related diseases [1]. Telomeres are specific DNA–protein structures found at the ends of chromosomes that preserve chromosomal stability and genomic integrity. Epidemiological studies have found significantly shorter telomeres in patients with coronary heart disease, with an inverse correlation according to the severity of the disease [2]. A meta-analysis of 24 prospective and retrospective studies reported a relative risk for coronary heart diseases (CAD) of 1.40 (1.15 to 1.70) in prospective studies and 1.80 (1.32 to 2.44) in retrospective ones, in patients with the shortest versus the longest tertiles of leukocyte telomere length (TL) [3]. Since TL was measured before the onset of the disease in the prospective ones, it supports the hypothesis that telomere shortening may cause CAD, rather than telomere shortening is a consequence of CAD. Furthermore, TL has been associated with dyslipidemia [4], familiar hypercholesterolemia [5], and hypertension [6], and it has been found shorter in type 2 diabetics (T2D) compared with their age-matched peers without T2D and also associated with more rapid diabetes progression [7].

TL is regulated by two opposing mechanisms: attrition that occurs in each cell division and elongation, that is modulated mainly, but not only, by the enzyme telomerase, which consists of a catalytic subunit telomerase reverse transcriptase (TERT), and an RNA component (TERC), which acts as a template. Furthermore, this telomere shortening is regulated by numerous additional proteins, such as WRAP53, all required to complete the complex telomerase repairing process [8]. While the relationship between TL and chronic diseases has been largely studied in observational studies of human populations, telomerase activity (TA) has only been approached in animal studies or in few small human cohorts. In this sense, TA was elevated in patients with unstable angina [9]. In addition, increased telomerase expression was detected in cardiomyocytes, endothelial cells, and fibroblasts of cryoinjured adult mice hearts, supporting the role of telomerase in regulating tissue repair and regeneration [10]. In contrast, an experimental study conducted in TERC^-^/^-^ mice found fewer atherosclerotic lesions development compared with their normal counterparts, suggesting that the absence of TA is protective for atherosclerotic disease [11]. However, the same knockout animal model exhibited a cardiac dysfunction similar to human cardiomyopathy, and the forced expression of *TERT* in cardiomyocytes promoted cell survival, thus supporting that TA could protect cardiac function [12]. These conflicting results suggest that the potential effect of telomerase strongly depends on the cell type and underlying pathology. How telomerase is regulated in leukocytes of patients with cardiovascular diseases (CVD) remains still unknown. 

Dyslipidemia is one of the most important risk factors for CVD, co-occurs with, and increases the risk of prediabetes and T2D [13,14], and both the concentration and the type of lipoproteins could underly these associations. Previous studies have briefly explored the potential correlations between lipoprotein particles size and TL. An analysis conducted in the framework of the NHANES study found a positive correlation between HDL cholesterol (HDL-C) and TL, whereas triglycerides were inversely associated [15]. A later re-analysis also conducted on the NHANES study displayed a non-linear relationship between HDL-C and TL, with a positive association when TL was less than 1.25 [16]. Since lipoprotein’s subfractions, defined by differences in particle size and density, improve the clinical assessment of CVD risk beyond standard lipid risk markers [17,18,19], we examine for the first time the associations of lipoprotein subfractions assessed via nuclear magnetic resonance (NMR) with leukocyte telomere length, *TERT*, and *WRAP53* expression, as surrogate indicators of telomerase activity [20,21] in pre-diabetic subjects free of CVD. 

## 2. Materials and Methods

### 2.1. Study Characteristics and Collection of Biological Samples 

This is a cross-sectional analysis within the framework of the EPIRDEM study, a randomized cross-over clinical feeding trial conducted over 54 pre-diabetic men and men and women, aged 25 to 65 years, with a body mass index (BMI) < 35 kg/m^2^. Subjects were excluded if they had diabetes or were using oral antidiabetic drugs; had an alcohol, tobacco, or drug abuse; were frequent consumers of nuts or had nut’s allergy; or were regular consumers of nutritional supplements, among others. Detailed information on the study can be found in Hernández-Alonso et al. [22]. All participants provided a written informed consent to participate in the study. The study protocol was approved by the institutional Ethical Committee in September 2011. The trial is registered in ClinicalTrials.gov (National Institutes of Health) with the identifier NCT01441921.

Medical data regarding concomitant diseases and medication were obtained from medical registers. Data on weight, height, waist circumference, BMI, body composition (Human-Im-Scan; Dietosystem, Barcelona, Spain), and blood pressure (HEM-705CP; OMRON, Hoofddorp, the Netherlands) were obtained using clinical standardized protocols. Physical activity was evaluated using the validated Spanish version of the Minnesota Leisure Time Physical Activity Questionnaire [23].

Plasma fasting glucose and serum lipid profile were determined using standard enzymatic automated methods, and LDL-C was estimated using the Friedewald equation in subjects with triglyceride levels lower than 400 mg/dL. Total blood and plasma samples were frozen at −80 °C for further analyses. 

### 2.2. Lipoprotein Analysis by NMR Spectroscopy of Plasma Samples

A previous methodology [24] was used to perform 2D diffusion-ordered ^1^H NMR spectroscopy (DOSY) lipoprotein analysis of plasma samples. For three different classes of lipoproteins—(I) VLDL (38.6–81.9 nm), (II) LDL (14.7–26.6 nm), and (III) HDL (6.0–10.9 nm)—sizes and particle numbers, as well as the particle numbers of nine subclasses (namely large, medium, and small VLDL, LDL, and HDL, respectively) were measured. A BrukerAvance III 600 spectrometer was used to record 2D ^1^H NMR spectra at 310 K (Bruker BioSpin, Rheinstetten, Germany). The bipolar gradient pulses of the double stimulated echo (DSTE) pulse program with a longitudinal eddy-current delay (LED) were employed. A total of 32 scans were recorded for each sample with a relaxation delay of 2 s, and the finite impulse decays (FIDs) were gathered into 64K complex data points. The gradient pulse strength was systematically increased from 5 to 95% of the maximum strength of 53.5 Gauss cm^−1^ in 32 steps. The pulse strength of the squared gradient followed a linear distribution. The nine lipoprotein subclasses were identified by surface fitting the methyl signal with the number of functions to calculate lipoprotein size. The mean particle size for each main fraction was calculated by averaging the NMR area of each fraction by its corresponding size. Each NMR region was divided by its corresponding volume to obtain particle-weighted lipoprotein sizes. By dividing the volume of the lipid by the volume of the particles belonging to a particular class, the particle numbers of each lipoprotein major fraction were determined. The lipid volumes were calculated by converting concentration units from partial least-squares (PLS) models into volume units using conventional conversion factors. The particle counts of the nine lipoprotein subclasses were derived from the relative areas of the lipoprotein components utilized to decompose the 2D spectra. The median and interquartile range of concentrations for the 12 quantified lipoproteins and subclasses are given in Table S1. 

### 2.3. Telomere Length Measurement 

DNA from whole blood samples was extracted with the Pure Link Genomic DNA (Invitrogen, Carlsbad, CA, USA) according to the manufacturer protocol. A previously published monochrome multiplex real-time quantitative PCR approach was used to quantify TL [25]. This method enables the simultaneous quantification of relative copy numbers of telomeres and single copy genes in a single reaction and calculates TL as a ratio of these two parameters. Each 384-well plate had a calibration curve with a reference DNA sample (1.50–2.34 ng/L in 2-fold dilutions) that was used for the relative quantification. The QuantiTect Syber Green PCR kit (QIAGEN, Hilden, Germany), telomere primer pairs, albumin primer pairs, and ultrapure water were included in the master mix to make up the entire final volume. The single-copy genes *albu* and *albd* (final concentration 900 nM each) were coupled with the primer pair telg and telc [26], each at a final concentration of 900 nM. All samples were tested in triplicate, and the experiment was carried out on a 384-well plate [26]. 

### 2.4. TERT and WRAP53 Expression

Total mRNA was isolated from whole-blood samples (Tempus Spin RNA Isolation Kit (Ambion Inc., Austin, TX, USA)) in accordance with the manufacturer’s instructions and retro-transcribed (High Capacity cDNA Reverse Transcription Kit (Invitrogen)) in accordance with the manufacturer’s instructions. cDNA was amplified using qPCR. *TERT* and *WRAP53* expression was normalized by the mean of *GAPDH* and *HPRT1* (TaqMan Fast Advanced Master Mix (Applied Biosystems) (Foster City, CA, USA)). 

### 2.5. Statistical Analysis 

Continuous data of study participants are presented depending on their distribution: normally distributed continuous data are presented as mean (standard deviation), while non-normally distributed continuous data are presented as median [interquartile range]. Categorical variables are presented as frequency and percentage (%). Initially, a thorough examination of the lipoproteins was conducted to ensure that an excessive proportion of missing values (>20%) was not present (Figure S1). All lipoproteins and lipoproteins subclasses assessed had an average percentage of missingness (min, max) equal to 2.16% (0.00, 11.11%). The random forest imputation method, using the “missForest” R package, was performed to impute the missing values of the lipoprotein subclasses in accordance with recommendations for metabolomics studies [27]. Lipoproteins were subjected to an inverse normal transformation, resulting in a rank-based standard normal distribution (mean = 0, SD = 1). 

For the three dependent variables—TL, *TERT*, and *WRAP53*—the 99th percentile of each outlier statistic was used as the cut-off point to identify significant outliers, resulting in the exclusion of 1 observation for each variable. A logarithmic base 10 transformation was applied to *TERT* and *WRAP53* to improve their distribution. Using a multiple imputation with chained equations by the R “MICE” package [28], we imputed their missing data. 

To determine a lipoprotein profile associated with TL, we regressed TL, *TERT*, and *WRAP53* on the 12 lipoproteins and lipoprotein subclasses. Given the dimensionality and the collinear nature of the data (Figure S2), we employed a Gaussian linear regression method with Lasso penalty to effectively address the issue of multicollinearity, using the “caret” R package [29]. Given the small sample size, we employed leave-one-out cross-validation (LOOCV). In each iteration, the Lasso method was implemented on N-1 samples as the training set, and the remaining case was used as a one-case validation set (intra-validation sets). A predictive model was subsequently trained by employing the estimated parameter and was blindly applied to the held-out sample. To account for the variability in the model’s performance due to the limited sample size, 25 repetitions of bootstrap aggregation were employed. Subsequently, the selected parameters were applied to the validation set, and the RMSE and Pearson correlation coefficients were calculated. 

To enhance stability in variable selection, the lipoprotein coefficients were obtained by averaging those obtained through LOOCV. Furthermore, only coefficients that were consistently present in at least 48 out of the 54 iterations (88.89%) were retained. Based on the available data, we calculated the 95% confidence interval. Finally, for each chosen lipoprotein particle, we determined the lipoprotein score (lipoprotein profile) as the weighted sum of the averaged coefficients from the 54 iterations. The effectiveness of the lipoprotein profile in determining its relation to telomere-related parameters was assessed using Pearson correlation coefficients between the reported and predicted. 

To address potential confounding effects of age (continuous), sex, BMI (continuous), dyslipidemia (yes, no), statins (yes, no), and physical activity leisure time (Kcal/day, continuous) on the association between lipoproteins and TL, *TERT*, and *WRAP53*, we conducted a sensitivity analysis by adding them as covariates. All analyses were performed using R version 4.2.1 (2022-06-23) (R Foundation for Statistical Computing, Vienna, Austria). 

## 3. Results

From a total of 54 participants allocated to the EPIRDEM study, nucleic acid samples were not available in 5 of them. Missing data were imputed using the methodological approach detailed in the Materials and Methods section. Participants’ characteristics are summarized in Table 1. Participants were middle age and pre-diabetic, with a mean BMI of 28.86 kg/m^2^. Furthermore, half of the participants had dyslipidemia. 

Lipoprotein particles descriptive analysis revealed an imbalance in the concentrations of different HDL particle sizes, with the concentration of S-HDL-P being up to 28 times higher than that of L-HDL-P. Furthermore, concentrations of the different LDL and VLDL particle sizes increased as the size decreased (Table S1). 

### Associations of Lipoproteins and Their Subclasses with TL, TERT, and WRAP53

**Figure 1:**
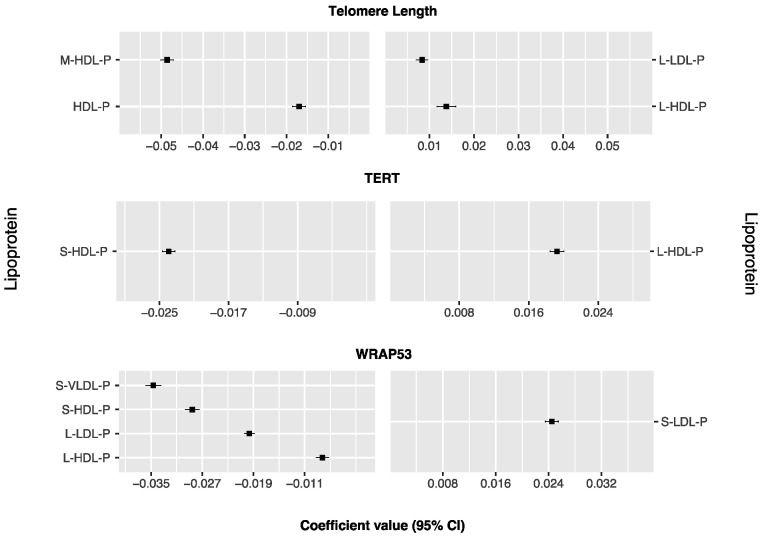
Lipoproteins ranked from highest to lowest Lasso positive and negative regression coefficients for telomere length, *TERT* (Telomerase Reverse Transcriptase), and *WRAP53* (WD Repeat Containing Antisense To TP53). Lipoproteins associated with negative coefficients (*n* = 7) are plotted on the left side, whereas those associated with positive coefficients (*n* = 4) are shown on the right side. The magnitude of the coefficient reflects the strength and direction of the association, with larger coefficients indicating stronger associations.

Figure 1 and Table S2 show different lipoproteins associated with TL, *TERT*, and *WRAP53* and selected 48 times in the LOOCV of the Lasso regressions. Large LDL and HDL particles were positively associated with TL, whereas total and medium HDL-P displayed inverse associations. Large HDL particles were positively associated with *TERT* expression, while medium HDL-P were negatively associated with *TERT* expression. For *WRAP53* expression, only small LDL particles were positively associated, whereas small and large HDL-P, S-VLDL-P, and L-LDL-P were inversely associated.

The Pearson correlation coefficients between TL, *TERT*, *WRAP53*, and their corresponding lipoprotein profiles were 0.350 (95% CI, 0.085 to 0.560, *p*-value = 0.011), 0.316 (95% CI, 0.052 to 0.538, *p*-value = 0.020), and 0.375 (95% CI, 0.120 to 0.584, *p*-value = 0.005), respectively (Table 2). 

Sensitivity analysis adjusting for potential covariates revealed consistent associations (Table S3). Medium HDL-P was negatively associated with TL. For *TERT* expression, large LDL particles were positively associated, and small HDL particles were inversely associated. Moreover, in contrast to the previous model, medium VLDL-P was negatively associated. The expression of *WRAP53* showed a positive correlation with small LDL particles but a negative correlation with small VLDL and HDL particles, as well as large LDL particles. 

## 4. Discussion

Using baseline data from the EPIRDEM study, we identified three unique lipoprotein profiles, all significantly correlated with TL, *TERT*, and *WRAP53*. Overall, small and medium HDL-P were inversely associated with longer telomeres and *TERT* expression but positively associated with *WRAP53*, whereas large HDL particles were positively associated with TL and *TERT* but negatively associated with *WRAP53* expression. 

Telomere length shortens in each cell division and is regularly repaired by telomerase which consists of two major components, TERT and TERC, and other proteins, such as WRAP53. Therefore, a reduction in *TERT* expression and a dysregulation of other telomerase-activity-related genes (i.e., *WRAP53*) may contribute to shortening telomeres [30]. Although the basis of shortening telomeres is not yet fully understood, TL and indeed telomerase activity are strongly influenced by inflammation and oxidation [31]. Previous metabolomic studies have suggested that lipid metabolism could play a key role in the regulation of TL. Indeed, several fatty-acid derived metabolites including glycerophosphoethanolamines, glycerophosphocholines, glycerolipids, phosphatidylcholines, and lysolipids have been associated with TL [32]. Furthermore, lipoproteins such as not only HDLc, but also total cholesterol and tryglycerides (TG) have been associated with TL [15,33]. A more recent study conducted in the framework of the NHANES 1999–2002 database revealed a non-linear positive association between HDLc and telomere length, which could be explained by the dysfunctionality of HDLc in pathological states such as inflammation [34]. No associations, neither with LDLc nor with TG, were found [16].

Low HDLc and high concentrations of LDLc have been strongly associated with a higher risk of CVD [35]. However, epidemiological evidence supports that their particles exist in a variety of sizes that may differentially affect the progression of CVD, with potential usefulness as clinical targets. Large HDL particles are associated with lower CVD risk in large-scale clinical studies [36], while small HDL particles typically reveal positive associations [36]. Regarding LDL particles of small and medium sizes, a positive association with CVD risk has been observed, suggesting that they may have atherogenic properties [37]. The relationship between lipoprotein particles with telomere length, although promising, has received very limited attention. A recent analysis of six independent population-based cohorts conducted on 11.775 subjects found a positive association between cholesterol to lipid ratios in small VLDL (S-VLDLc % and S-VLDLce %) and cholesterol esters in very small VLDL (XS-VLDLce) with TL. Furthermore, L-VLDL-P are rich in TG [38] and have been associated with incident hypertension [39]. These results suggest a complex interaction between lipid metabolism and telomere shortening and highlight that lipoprotein particle size and subclasses drive the associations with TL, and not the lipoproteins analyzed using clinical assays [40,41]. In contrast, we failed to find associations between VLDL particles, TL, and *TERT*, but we found an inverse association between small VLDL particles and *WRAP53* expression, one of the proteins involved in the telomerase trafficking and assembly, that would apparently reduce the telomerase activity [42]. Whether this VLDL particle is associated with *WRAP53* via atherogenic effects is unknown, and further research is needed to elucidate this issue [43]. 

We found large HDL particles positively associated with telomere length and *TERT* expression. On the other hand, medium and total HDL particles were inversely associated with TL, whereas small-sized HDL particles were inversely associated with *TERT*. Small HDL particles have been positively associated with incident hypertension in a prospective cohort study of 17,527 initially healthy women followed for 8 years [39]. Furthermore, shorter TL has been related to hypertension [6], and this association is further strengthened in the presence of insulin resistance [44]. These results suggest that HDL particles may have important implications in age-related diseases. 

Despite the considerable attention given to the role of HDL in cardiovascular risk assessment, the relationship between these two factors remains controversial [45]. Smaller HDL particles’ potent antioxidative activity could be compromised by atherogenic dyslipidemia, as evidenced by previous studies [46], which have also shown an inverse correlation between HDL particle size and the risk of developing coronary heart disease [47]. Although the association of the total HDL with TL is somehow unexpected, this relationship could be driven by small HDL since among all HDL particles it is the one that was found in higher concentration in our study population. 

Moreover, small-sized and large HDL particles were inversely associated with *WRAP53* expression. Mutations in *WRAP53* gene results in shortening telomeres [48] and reduced expression in ovarian tumors has been correlated with attenuated DNA damage response, since the protein WRAP53β rapidly accumulates at DNA breaks and recruits DNA repair proteins [49]. However, the complex functions of this protein do not allow us to necessarily assume that a leukocyte reduction in *WRAP53* expression substantially reduces the activity of the telomerase complex leading to shorter telomeres [50]. 

Large-sized LDL particles were positively associated with TL and inversely associated with *WRAP53*. A recent longitudinal study of metabolomic data from 1162 participants within the Hortega Study [37] identified that an increase in medium-sized LDL and small-sized LDL and a decrease in L-LDL particles had a significant association with coronary heart disease, corroborating our results. Nevertheless, we found a direct association between S-LDL-P and *WRAP53* gene expression. 

To the best of our knowledge, this is the first study evaluating, in the same study participants, the associations between leukocyte telomere length and the expression of *TERT* and *WRAP53* with the size of lipoprotein particles assessed via a robust NMR-based metabolic platform. The study has some limitations, including the relatively small sample size that could limit the statistical power for additional findings. Another limitation is that participants included in this analysis were pre-diabetic, which makes it difficult to generalize our findings. Replication of the finding in an independent study with a large sample size, and wider analyses of telomerase complex will strengthen the study conclusions. Longitudinal data and a more detailed approach for covering telomerase complex and activity might provide additional and valuable information into telomere length and lipid profile interaction, and a possible reverse causation. 

## 5. Conclusions

In summary, we found several lipoprotein particles associated with TL, *TERT*, and *WRAP53*. Overall, total and medium HDL particles were associated with shorter telomeres and lower expression of *TERT* and *WRAP53*. Large HDL particles were associated with longer telomeres, and higher *TERT* expression but lower expression of *WRAP53*. Despite these findings, the underlying mechanisms are not fully understood, and further research is needed to elucidate the relationship between lipid profile and telomeres, as well as its potential clinical implications. 

## Figures and Tables

**Table 1:** Baseline characteristics of the study population.

Variable	Subjects (*n* = 54)
Age, years	55 (6.25)
Female, *n* (%)	25 (46)
Weight, kg	75.70 [71.00, 82.15]
Body mass index, kg/m^2^	28.86 (2.55)
Dyslipidemia, *n* (%)	27 (50)
Acetylsalicylic Acid, *n* (%)	3 (0.01)
Statins, *n* (%)	5 (9.3)
Leisure-time physical activity (Kcal/day)	347.40 (145.33)
Fasting plasma glucose (mg/dL)	110.33 (4.28)
Telomere length	0.76 [0.63, 0.88]
*TERT*	8.57 [7.37, 11.05]
*WRAP53*	3.46 [2.93, 3.72]

Normal distributed continuous data are presented as a mean (standard deviation); not normal distributed continuous data are presented as median [interquartile range]; and finally, for categorical variables, data are presented as frequency percentage (%).

**Table 2:** Pearson correlation coefficients of telomere length, *TERT* (Telomerase Reverse Transcriptase), and *WRAP53* (WD Repeat Containing Antisense To TP53) with the corresponding lipoprotein profiles.

Metabolites	r (95% CI)	*p*-Value
Telomere length	0.347 (0.088, 0.563)	0.010 *
*TERT*	0.316 (0.052, 0.538)	0.020 *
*WRAP53*	0.379 (0.124, 0.587)	0.005 **

* *p* value < 0.05, ** *p* value < 0.01.

## Data Availability

This study is registered at www.clinicaltrials.gov as NCT01441921. It was accessed in December 2018. Data will be available under request.

## Supplementary Materials

The following supporting information can be downloaded at https://www.mdpi.com/article/10.3390/nu15112624/s1. Figure S1: Plot of the 12 lipoproteins subclasses according to the percentage of missingness in 54 participants; Figure S2: Spearman correlation matrix for all the 12 lipoproteins and lipoprotein subclasses considered in the analysis; Table S1: Median and interquartile range of concentrations for the 12 lipoproteins subclasses quantified by nuclear magnetic resonance; Table S2: List of lipoproteins subclasses selected at least 90% times in the leave-one-out cross-validation; Table S3: Lipoprotein subclasses ranked from highest to lowest LASSO positive and negative regression coefficients for telomere length, *TERT* (Telomerase Reverse Transcriptase), and *WRAP53* (WD Repeat Containing Antisense To TP53) in a sensitivity analysis with adjustments.
