# Supplementary material for: Lipoprotein Particle Profiles Associated with Telomere Length and Telomerase Complex Components
Source: Nutrients. 2023 Jun 3;15(11):2624. doi: 10.3390/nu15112624 (PMC10255183; doi:10.3390/nu15112624)
Supplement: Supplementary file 1 [file nutrients-15-02624-s001.zip › nutrients-2415625-supplementary.pdf]

Supplementary information for

Lipoprotein particle profiles associated with telomere length and telomerase complex components.

Nil Novau-Ferré [1,2,3], Melina Rojas [1,2,3], Laia Gutierrez-Tordera [1,2,3], Pierre Arcelin [2,4], Jaume Folch [1,2,3,5], Christopher Papandreou [1,2,3,6,*,†] and Mònica Bulló [1,2,3,4,7,*,†]

1 Nutrition and Metabolic Health Research Group, Department of Biochemistry and Biotechnology, Rovira i Virgili University (URV), 43201 Reus, Spain; nil.novau@urv.cat (N.N.-F.); melinaisabella.rojas@urv.cat (M.R.); laia.gutierrez@iispv.cat (L.G.-T.); jaume.folch@urv.cat (J.F.); monica.bullo@urv.cat (M.B.)
2 Institute of Health Pere Virgili (IISPV), Reus, Spain. Institute of Health Pere Virgili (IISPV), 43204 Reus, Spain; pierrealphonse.arcelin@salutsantjoan.cat (P.A.); christoforos.papandreou@iispv.cat (C.P.)
3 Center of Environmental, Food and Toxicological Technology (TecnATox), Rovira i Virgili University, 43201 Reus, Spain.
4 Atención Basica de Salut (ABS) Reus V. Centro de Atención Primaria Marià Fortuny, SAGESSA, 43204 Reus, Spain.
5 Biomedical Research Networking Centre in Neurodegenerative Diseases (CIBERNED), Carlos III Health Institute, 28031 Madrid, Spain.
6 Department of Nutrition and Dietetics Sciences, School of Health Sciences, Hellenic Mediterranean University (HMU), 72300 Siteia, Greece
7 CIBER Physiology of Obesity and Nutrition (CIBEROBN), Carlos III Health Institute, 28029 Madrid, Spain.
* Correspondence: christoforos.papandreou@iispv.cat (C.P.); monica.bullo@urv.cat (M.B.)
† Senior authors.

This PDF file includes:

Figures S1 to S2
Tables S1 to S3

Table S1. Median and interquartile range of concentrations for the 12 lipoproteins subclasses quantified by nuclear magnetic resonance.

Metabolites	Median [interquartile range]
Total VLDL-P (nM)	36.06 [25.46, 53.06]
L-VLDL-P (nM)	0.80 [0.48, 1.46]
M-VLDL-P (nM)	4.62 [2.62, 7.86]
S-VLDL-P (nM)	31.29 [22.79, 46.22]
Total LDL-P (nM)	1230.05 [1052.30, 1356.00]
L-LDL-P (nM)	133.03 [105.87, 164.97]
M-LDL-P (nM)	455.33 [374.00, 526.50]
S-LDL-P (nM)	609.77 [519.80, 695.60]
Total HDL-P (μM)	30.65 [27.92, 33.11]
L-HDL-P (μM)	0.81 [0.43, 1.41]
M-HDL-P (μM)	6.44 [4.52, 7.84]
S-HDL-P (μM)	22.85 [20.58, 24.85]

Abbreviations: -P, particle; L-, Large; M-, Medium; S-, Small; HDL, high-density lipoprotein; LDL, low-density lipoprotein; VLDL, very low-density lipoprotein.

Table S2. List of lipoproteins subclasses selected at least 90% times in the leave-one-out cross-validation.

**Telomere length**			
**Lipoproteins subclasses**	**β coefficient (95% CI)**	**Lipoproteins subclasses**	**β coefficient (95% CI)**
L-HDL-P	0.014 (0.012, 0.016)	M-HDL-P	-0.049 (-0.050, -0.047)
L-LDL-P	0.008 (0.007, 0.010)	HDL-P	-0.017 (-0.019, -0.015)
TERT			
**Lipoproteins subclasses**	**β coefficient (95% CI)**	**Lipoproteins subclasses**	**β coefficient (95% CI)**
L-HDL-P	0.019 (0.019, 0.020)	S-HDL-P	-0.024 (-0.025, -0.023)
WRAP53			
**Lipoproteins subclasses**	**β coefficient (95% CI)**	**Lipoproteins subclasses**	**β coefficient (95% CI)**
S-LDL-P	0.025 (0.024, 0.026)	S-VLDL-P	-0.035 (-0.036, -0.034)
		S-HDL-P	-0.029 (-0.030, -0.028)
		L-LDL-P	-0.020 (-0.020, -0.019)
		L-HDL-P	-0.008 (-0.009, -0.007)

Abbreviations: -C, cholesterol; -P, particle; L-, Large; M-, Medium; S-, Small; HDL, high-density lipoprotein; LDL, low-density lipoprotein; VLDL, very low-density lipoprotein; %, Percentage; CI, confidence interval.

Figure S1. Plot of the 12 lipoproteins subclasses according to the percentage of missingness in 54 participants.

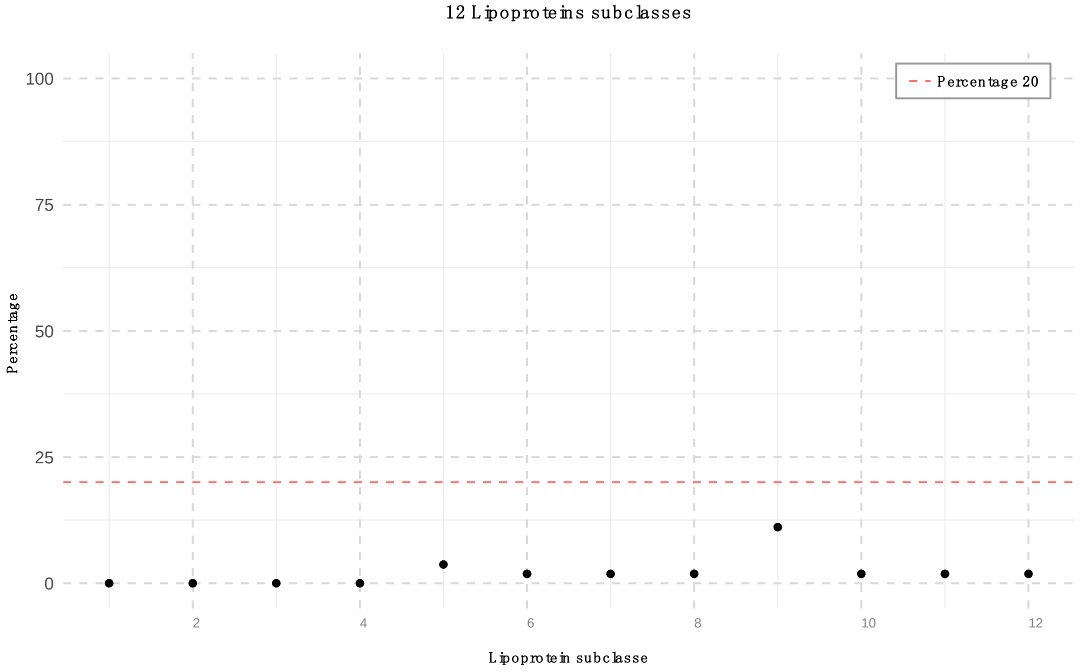

Figure S2. Spearman correlation matrix for all the 12 lipoproteins and lipoprotein subclasses considered in the analysis. Spearman correlation matrix for all the 12 lipoproteins and lipoprotein subclasses considered in the analysis. The colors in the matrix represent the direction of the correlation, with blue indicating a positive correlation and red indicating an inverse correlation. The color depth corresponds to the magnitude of the correlation, with darker shades indicating stronger correlations. Significant correlations are indicated by dots.

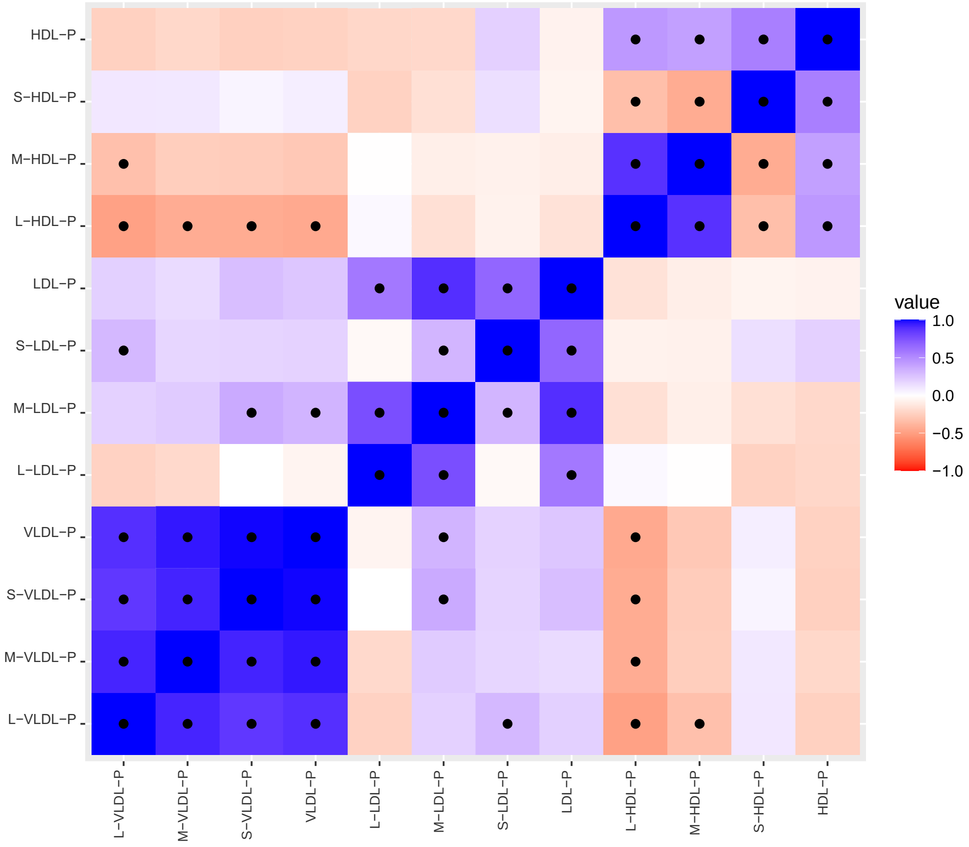

Table S3. Lipoprotein subclasses ranked from highest to lowest LASSO positive and negative regression coefficients for telomere length, *TERT* (Telomerase Reverse Transcriptase), and *WRAP53* (WD Repeat Containing Antisense To TP53) in a sensitivity analysis with adjustments.

**Telomere length**			
**Lipoproteins subclasses**	**β coefficient (95% CI)**	**Lipoproteins subclasses**	**β coefficient (95% CI)**
		M-HDL-P	-0.020
TERT			
**Lipoproteins subclasses**	**β coefficient (95% CI)**	**Lipoproteins subclasses**	**β coefficient (95% CI)**
L-HDL-P	0.011	S-HDL-P	-0.014
		M-VLDL-P	-0.002
WRAP53			
**Lipoproteins subclasses**	**β coefficient (95% CI)**	**Lipoproteins subclasses**	**β coefficient (95% CI)**
S-LDL-P	0.019	S-VLDL-P	-0.038
		S-HDL-P	-0.023
		L-LDL-P	-0.016

Abbreviations: -P, particle; L-, Large; M-, Medium; S-, Small; HDL, high-density lipoprotein; LDL, low-density lipoprotein; VLDL, very low-density lipoprotein.

Regression was adjusted for age (continuous), sex, BMI (continuous), dyslipidemia, acetylsalicylic acid consumption, statin use, and leisure-time physical activity (measured in Kcal/day) (continuous).
